# Rates and correlates of DSM-5 mixed features among individuals with affective disorders: a cross-sectional study

**DOI:** 10.1192/j.eurpsy.2023.1077

**Published:** 2023-07-19

**Authors:** F. Bartoli, D. Palpella, T. Callovini, B. Bachi, R. Cioni, F. Moretti, A. Canestro, M. Morreale, S. Piacenti, A. Bartoccetti, M. Castiglioni, S. Limonta, M. Re, C. Crocamo, G. Carrà

**Affiliations:** 1 Department of Medicine and Surgery, University of Milano Bicocca, Milano, Italy; 2 Division of Psychiatry, University College London, London, United Kingdom

## Abstract

**Introduction:**

The definition of mixed states has been changed over the years, leading to substantial heterogeneity and inconsistencies across studies, and thus limiting the understanding of this phenomenon. Given the limited data available after the introduction of the DSM-5 mixed features specifier (MFs), we conducted a cross-sectional study evaluating MFs in individuals suffering from affective disorders, i.e., major depressive disorder (MDD) or bipolar disorder (BD).

**Objectives:**

The aim of this study is to evaluate rates and correlates of MFs in a consecutive sample of inpatients with mood episodes.

**Methods:**

We included adults consecutively admitted to our inpatient mental health unit with a current manic episode (ME) or major depressive episode (MDE). DSM-5 criteria were used to assess the occurrence of MFs. Young Mania Rating Scale (YMRS) and Montgomery-Åsberg Depression Rating Scale (MADRS) were used to assess the severity of the mood episodes. We used the Kemp Compliance Rating Scale to assess medication adherence.

**Results:**

A total of 285 individuals were included (mean age ± SD: 48.3 ± 17.9; M/F ratio: 2/3). Among them, 94 (33.0%) were in a ME and 191 (67.0%) in a MDE. Forty individuals (14.0%) exhibited MFs. We found that MFs were significantly more frequent in participants with a diagnosis of BD (p<0.001) and during a ME (p=0.006). In addition, study participants with MFs had lower medication adherence at hospital admission (p=0.008). Finally, individuals with ME and MFs had lower YMRS scores than those without MFs (p<0.001), and, similarly, those with MDE and MFs had lower MADRS scores than those without MFs (p<0.001).

**Conclusions:**

Considering DSM-5 classification, we found that MFs are a phenomenon strongly linked to BD. While the symptom severity of the prevalent polarity tends to be lower in episodes with MFs, the reduced adherence may be suggestive of a more complex clinical management requiring specific treatment approaches.

**Disclosure of Interest:**

None Declared

